# Transcriptome profiling of intact bowel wall reveals that PDE1A and SEMA3D are possible markers with roles in enteric smooth muscle apoptosis, proliferative disorders, and dysautonomia in Crohn’s disease

**DOI:** 10.3389/fgene.2023.1194882

**Published:** 2023-08-31

**Authors:** Yun Yang, Lin Xia, Wenming Yang, Ziqiang Wang, Wenjian Meng, Mingming Zhang, Qin Ma, Junhe Gou, Junjian Wang, Ye Shu, Xiaoting Wu

**Affiliations:** ^1^ Department of General Surgery, West China Hospital, Sichuan University, Chengdu, China; ^2^ Colorectal Cancer Center, West China Hospital, Sichuan University, Chengdu, China; ^3^ Department of General Surgery, West China Chengdu Shangjin Nanfu Hospital, Sichuan University, Chengdu, China; ^4^ Division of Gastrointestinal Surgery, Department of General Surgery, West China Hospital, Sichuan University, Chengdu, China; ^5^ Department of Pathology, West China Hospital, Sichuan University, Chengdu, China; ^6^ Department of Laboratory Medicine, West China Hospital, Sichuan University, Chengdu, China; ^7^ Colorectal and Pelvic Floor Center, West China Tianfu Hospital, Sichuan University, Chengdu, China

**Keywords:** Crohn’s disease, RNA seq, autonomic nervous system, smooth muscle cell, proliferation, apoptosis, Sema3D, PDE1A

## Abstract

**Background:** Inflammatory bowel disease (IBD) is a complex and multifactorial inflammatory condition, comprising Crohn’s disease (CD) and ulcerative colitis (UC). While numerous studies have explored the immune response in IBD through transcriptional profiling of the enteric mucosa, the subtle distinctions in the pathogenesis of Crohn’s disease and ulcerative colitis remain insufficiently understood.

**Methods:** The intact bowel wall specimens from IBD surgical patients were divided based on their inflammatory status into inflamed Crohn’s disease (iCD), inflamed ulcerative colitis (iUC) and non-inflamed (niBD) groups for RNA sequencing. Differential mRNA GO (Gene Ontology), and KEGG (Kyoto Encyclopedia of Genes and Genomes), and GSEA (Gene Set Enrichment Analysis) bioinformatic analyses were performed with a focus on the enteric autonomic nervous system (ANS) and smooth muscle cell (SMC). The transcriptome results were validated by quantitative polymerase chain reaction (qPCR) and immunohistochemistry (IHC).

**Results:** A total of 2099 differentially expressed genes were identified from the comparison between iCD and iUC. Regulation of SMC apoptosis and proliferation were significantly enriched in iCD, but not in iUC. The involved gene PDE1A in iCD was 4-fold and 1.5-fold upregulated at qPCR and IHC compared to that in iUC. Moreover, only iCD was significantly associated with the gene sets of ANS abnormality. The involved gene SEMA3D in iCD was upregulated 8- and 5-fold at qPCR and IHC levels compared to iUC.

**Conclusion:** These findings suggest that PDE1A and SEMA3D may serve as potential markers implicated in enteric smooth muscle apoptosis, proliferative disorders, and dysautonomia specifically in Crohn’s disease.

## 1 Introduction

Inflammatory bowel disease (IBD) is a chronic idiopathic inflammatory disorder characterized by relapsing and remitting symptoms. The two most common forms of IBD are Crohn’s disease (CD) and ulcerative colitis (UC) (Assadsangabi et al., 2019). Morphologically, UC primarily affects the rectum and colon, exhibiting superficial inflammation confined to the mucosal and submucosal layers, often accompanied by cryptitis and crypt abscesses. In contrast, CD is characterized by a non-continuous and transmural pattern of inflammation, presenting additional complications such as thickened submucosa and muscularis propria, intestinal fibrosis, strictures, fissuring ulceration, non-caseating granulomas, abscesses, and fistulas (Abraham and Cho, 2009). A notable feature of CD is the presence of fibrostenosis, which contributes to therapeutic challenges and the need for surgical resection. However, a recent histological grading scheme study discovered that smooth muscle hyperplasia/hypertrophy, rather than fibrosis, is the primary change associated with the “fibrostenosis” phenotype in CD. Neuromuscular hyperplasia/hypertrophy was also identified as a significant change (Chen et al., 2017).

Although morphological and histological differences exist between CD and UC, a comprehensive whole-genome gene expression meta-analysis (Granlund et al., 2013) based on 11 available datasets (Wu et al., 2007; Galamb et al., 2008a; Ahrens et al., 2008; Galamb et al., 2008b; Carey et al., 2008; Kugathasan et al., 2008; Noble et al., 2008; Arijs et al., 2009; Olsen et al., 2009; Bjerrum et al., 2010; van Beelen Granlund et al., 2013) did not unveil any significant differences between CD and UC. Interestingly, gene expression in the inflamed mucosa from both UC and CD was remarkably similar. The patterns of antimicrobial peptide (AMP) and T-helper cell-related gene expression were also comparable, except for the higher expression of IL23A observed in UC compared to CD. Another study conducted by the IBD-CHARACTER consortium, which included 323 subjects, found that a comparison of inflamed UC and inflamed CD identified 204 highly differentially expressed upregulated transcripts and 58 downregulated transcripts (Consortium, 2021). These two gene expression signatures were highly correlated, suggesting that inflammation might mask underlying biological differences among the diagnostic groups. Furthermore, when comparing inflamed biopsies from UC and CD on a biological pathway level, the normalized enrichment scores were remarkably similar, irrespective of diagnosis or whether healthy or symptomatic controls were used in the comparison. However, mitochondria-associated pathways exhibited negative normalized enrichment scores in inflamed UC compared to inflamed CD (Vatn et al., 2022).

Despite these findings, previous studies (Wu et al., 2007; Galamb et al., 2008a; Ahrens et al., 2008; Galamb et al., 2008b; Carey et al., 2008; Kugathasan et al., 2008; Noble et al., 2008; Arijs et al., 2009; Olsen et al., 2009; Bjerrum et al., 2010; van Beelen Granlund et al., 2013; Vatn et al., 2022) encountered limitations due to the challenges of obtaining surgical resection specimens. Instead, mucosa-submucosa (SM) specimens from colonoscopy pinch biopsies were commonly used. However, these specimens lack the layers of muscularis propria (MP) and subserosal adventitia (SS), making it difficult to fully elucidate the underlying disease-inducing mechanisms in IBD. Consequently, subtle differences between CD and UC might have been unintentionally overlooked.

Therefore, the present study aims to utilize intact bowel wall specimens obtained during surgical resection. Through RNA-seq, bioinformatics analysis, and validation using quantitative polymerase chain reaction (qPCR) and immunohistochemistry (IHC), we aim to explore the subtle differences between CD and UC, with a primary focus on smooth muscle cells (SMCs) and the enteric autonomic nervous system (ANS).

## 2 Materials and methods

### 2.1 Specimen collection

All the intact bowel wall specimens were collected from the Biobank of West China Hospital (WCH), Sichuan province, China. The study was approved (No. 20221470) and supervised by the WCH Ethics Committee. Patients who received bowel resection after being diagnosed with IBD were recruited. Informed consent was obtained from all patients in the study prior to the medical history and collection of specimens. For the inflamed CD (iCD) and inflamed UC (iUC) groups, specimens from the most inflamed segment within the colon were selected. For the non-inflamed (niBD) group, specimens from the uninvolved non-inflamed (niCD/niUC) segment within the colon were selected. The postoperative pathological diagnosis was confirmed by a team of pathologists using the guidelines on the pathological diagnosis of IBD (Shen and Weber, 2017).

### 2.2 RNA extraction and library preparation

Total RNA was extracted using TRIzol reagent (Cat.# 15596018, Thermo Fisher Scientific, United States of America) according to the manufacturer’s protocol. RNA purity and quantification were evaluated on the NanoDrop 2000 spectrophotometer (Thermo Fisher Scientific). RNA integrity was evaluated using the Agilent 2100 Bioanalyzer (Agilent Technologies, United States of America). The specimens with RNA integrity number (RIN) ≥7 were subjected to the subsequent analysis. The libraries were constructed using TruSeq Stranded Total RNA with Ribo-Zero Gold (Cat.# RS-122–2301, Illumina, United States of America) according to the manufacturer’s instructions and sequenced on the Illumina HiSeq X Ten platform; 150-bp paired-end reads were generated. The sequencing and analyses were performed by OE Biotech Co., Ltd. (Shanghai, China).

### 2.3 Bioinformatics analysis

Raw reads for each specimen were generated in FASTQ format and processed using the Trimmomatic software (Bolger et al., 2014). Subsequently, clean reads were obtained by removing the adapter and ploy-N or low-quality sequences from raw data. Then, the clean reads for each specimen were mapped to the human genome (GRCh38) using HISAT2(Kim et al., 2015). For mRNAs, FPKM (fragments per kilobase of exon model per million mapped fragments) (Roberts et al., 2011) of each gene was calculated using Cufflinks (Trapnell et al., 2010), and the read counts of each gene were obtained by HTSeq-count (Anders et al., 2015). Differential expression analysis was performed using DESeq (2012) R package (Anders et al., 2012). *p*-value <0.05 was set as the threshold for a significantly differential expression. The differential mRNA GO (Ashburner et al., 2000, 2021) and KEGG (Kanehisa et al., 2010; Kanehisa et al., 2017) enrichments were analyzed based on selected differential transcripts with *p*-values <0.05 and fold-change (FC) > 1.5 based on the hypergeometric distribution test. Also, gene set expression analysis (GSEA) of molecular pathways affected by differentially expressed genes (DEGs) was performed by GSEA R (v1.2) with weighted enrichment statistic and Signal2Noise for gene ranking (Mootha et al., 2003; Subramanian et al., 2005).

The data of gene sets analyzed on GSEA are summarized in Supplementary Tables S1 and S2.

### 2.4 Quantitative polymerase chain reaction (qPCR)

Quantification was performed with a two-step reaction: reverse transcription and PCR. Each 10-μL reaction of reverse transcription consisted of 0.5 μg RNA, 2 μL of 5× TransScript All-in-one SuperMix for qPCR, and 0.5 μL of gDNA Remover. The reactions were performed on a GeneAmp^®^ PCR System 9700 (Applied Biosystems, United States of America) at 42°C for 15 min and 85°C for 5 s. The 10-μL RT reaction mix was then diluted in 90 μL nuclease-free water and held at −20°C. Real-time PCR was performed on LightCycler^®^ 480 II Real-time PCR Instrument (Roche, Swiss) in a 10-μL PCR reaction mixture in a 384-well optical plate (Roche, Swiss), consisting of 1 μL of cDNA, 5 μL of 2× PerfectStartTM Green qPCR SuperMix, 0.2 μL of 10 µM forward primer, 0.2 μL of 10 µM reverse primer, and 3.6 μL of nuclease-free water. The reactions were incubated at 94°C for 30 s, followed by 45 cycles of 94°C for 5 s, and 60°C for 30 s. Each sample was assessed in triplicate. Finally, melting curve analysis was performed to validate the specific qPCR product. The expression levels of mRNAs were normalized to GAPDH. The primer sequences were designed in the laboratory and synthesized by TsingKe Biotech (Beijing, China), based on the mRNA sequences obtained from the NCBI database (Table 1).

**TABLE 1:** Primer sequences.

Num	Gene symbol	Direction	Primer sequences	Product length (bp)	Tm (°C)
1	SEMA3D	Forward	GTT​CAT​CAG​AAG​GAC​TGG​ATT	89	60
		Reverse	TAG​AAA​GAT​GTG​GTC​TTT​GGC		
2	SLC18A2	Forward	GAT​TTC​CAT​GGC​TCA​TGA​CA	89	60
		Reverse	TTCTTTGGCAGGTGGACT		
3	PDE1A	Forward	AAG​CAA​GTG​GAG​AGC​ATA​G	85	60
		Reverse	ACA​GGA​ATC​TTG​AAA​CGG​T		
4	TACR1	Forward	GAG​AAA​TAG​GAG​TTG​CAG​GC	84	60
		Reverse	AAG​AAA​TTC​CAC​CGG​TCA​C		
5	SPHK1	Forward	ACC​ATT​ATG​CTG​GCT​ATG​AG	96	60
		Reverse	GCAGGTTCATGGGTGACA		
6	ADRA1A	Forward	GTG​AAC​ATT​TCC​AAG​GCC​A	81	60
		Reverse	CAC​TAG​GAT​GTT​ACC​CAG​C		
7	GAPDH	Forward	CCT​CAC​AGT​TGC​CAT​GTA​GA	69	60
		Reverse	TGG​TAC​ATG​ACA​AGG​TGC​G		

### 2.5 Immunohistochemistry (IHC)

The expression of SEMA3D and PDE1A was assessed by IHC using formalin-fixed paraffin-embedded (FFPE) tissue. The staining antibodies were as follows: SEMA3D (dilution 1/50; Cat.# NBP1-85517, NOVUS, Centennial, United States of America) and PDE1A (dilution 1/200; Cat.# 12442-2-AP, Proteintech, Wuhan, China) (Supplementary Table S3). Antibody detection and visualization were performed using DAB (3,3′-diaminobenzidine) as the chromogenic substrate. The images were captured under BA400 Digital microscope (Motic, China). The percentage of DAB-positive tissue in each image was calculated using the Halo data analysis system (Halo 101-WL-HALO-1, Indica labs, United States of America).

### 2.6 Statistical analysis

Contingency data were assessed for significant differences using chi-square or Fisher’s exact test. The data were expressed as means ± standard deviation (SD). The comparison between the two groups was assessed using the Holm-Šídák test. The multiple comparisons were evaluated using Fisher’s LSD (least significant difference) test. *p*-value <0.05 indicated a statistically significant difference. The statistical analyses were performed using GraphPad Prism9 (GraphPad Software, United States of America).

## 3 Results

### 3.1 Demographic characteristics

The demographic and clinical information of the individual patient are summarized in Supplementary Table S4, and the grouping design is provided in Supplementary Table S5.

Both CD and UC specimens were divided based on inflammatory status into inflamed (iCD and iUC) and non-inflamed (niBD: niCD + niUC) groups.The total number of specimens primarily included 6 iCD, 6 iUC, and 6 niBD for RNA extraction and quality control. Since bacteria RNA contamination was detected in iCD4, iUC6 and niBD6 (Supplementary Figure S1), these 3 samples were excluded, the final total number of 15 specimens including 5 iCDs (iCD1, iCD2, iCD3, iCD5 and iCD6), 5 iUCs(iUC1, iUC2, iUC3, iUC4 and iUC5), and 5 niBDs (niBD1, niBD2, niBD3, niBD4, niBD5)were used for the subsequent bioinformatics analysis.

Overall, patients with UC had greater left colon (descending colon or rectal) involvement (*p* = 0.0152). Patients with CD tended to have a young onset age (iCD vs. iUC, 28.83 ± 9.06 vs. 64 ± 7.80 years old, *p* < 0.0001) and ileal involvement (*p* = 0.0152) and required the postoperative biological therapy. Importantly, unlike iUC, iCD had a higher depth score of inflammatory infiltration (iCD vs. iUC, 3.67 ± 0.52 vs. 2.00 ± 1.10, *p* = 0.0071) (Table 2).

**TABLE 2:** Demographic characteristics.

	iCD (n = 6)	iUC (n = 6)	niBD (n = 6)	*p*-value
Patient characteristics				
Age (years)	28.83 ± 9.06	64.00 ± 7.80	42.67 ± 18.69	0.0009*
Gender	6M 0F	5M 1F	5M 1F	0.5698
Smoker	1/6	3/6	2/6	0.4724
Alcohol	0/6	4/6	2/6	0.0498*
Preoperative treatment history				
5-ASA	4/6	6/6	4/6	0.2765
Steroids	3/6	4/6	3/6	0.7985
Immunomodulation	2/6	2/6	2/6	0.9999
Anti-TNF	2/6	0/6	0/6	0.1054
Non-anti-TNF biologic treatment	NA	NA	NA	NA
Location involvement				
Ileum	5/6	0/6	NA	0.0152*
Cecum	3/6	0/6	NA	0.1818
Ascending colon	3/6	5/6	NA	0.5455
Transverse colon	3/6	6/6	NA	0.1818
Descending colon	1/6	6/6	NA	0.0152*
Sigmoid	2/6	5/6	NA	0.2424
Rectal	0/6	5/6	NA	0.0152*
Phenotypes				
Depth score of inflammatory infiltration^※^	3.67 ± 0.52	2.00 ± 1.10	NA	0.0071*
Acute inflammation	2/6	4/6	NA	0.5671
Chronic inflammation	6/6	5/6	NA	0.9999
Ulcers	2/6	5/6	NA	0.2424
Penetrate/fistula	2/6	0/6	NA	0.4545
Stricturing	4/6	0/6	NA	0.0606
Periganglitis	2/6	0/6	NA	0.4545
Postoperative outcomes				
Biologic use	3/6	0/6	1/6	0.1054
Median time to first resection (months)	54.33 ± 45.86	53.08 ± 54.42	53.00 ± 45.29	0.9986
Median time from first resection to second resection (months)	NA	NA	NA	NA

^※^Depth score of inflammatory infiltration. Mucosa: Score 1; muscularis mucosa (MM): Score 2; submucosa (SM): Score 3; muscularis propria (MP): Score 4; subserosal adventitia (SS): Score 5.

### 3.2 Transcriptome profiling distinguished the differences between iCD and iUC

**FIGURE 1:**
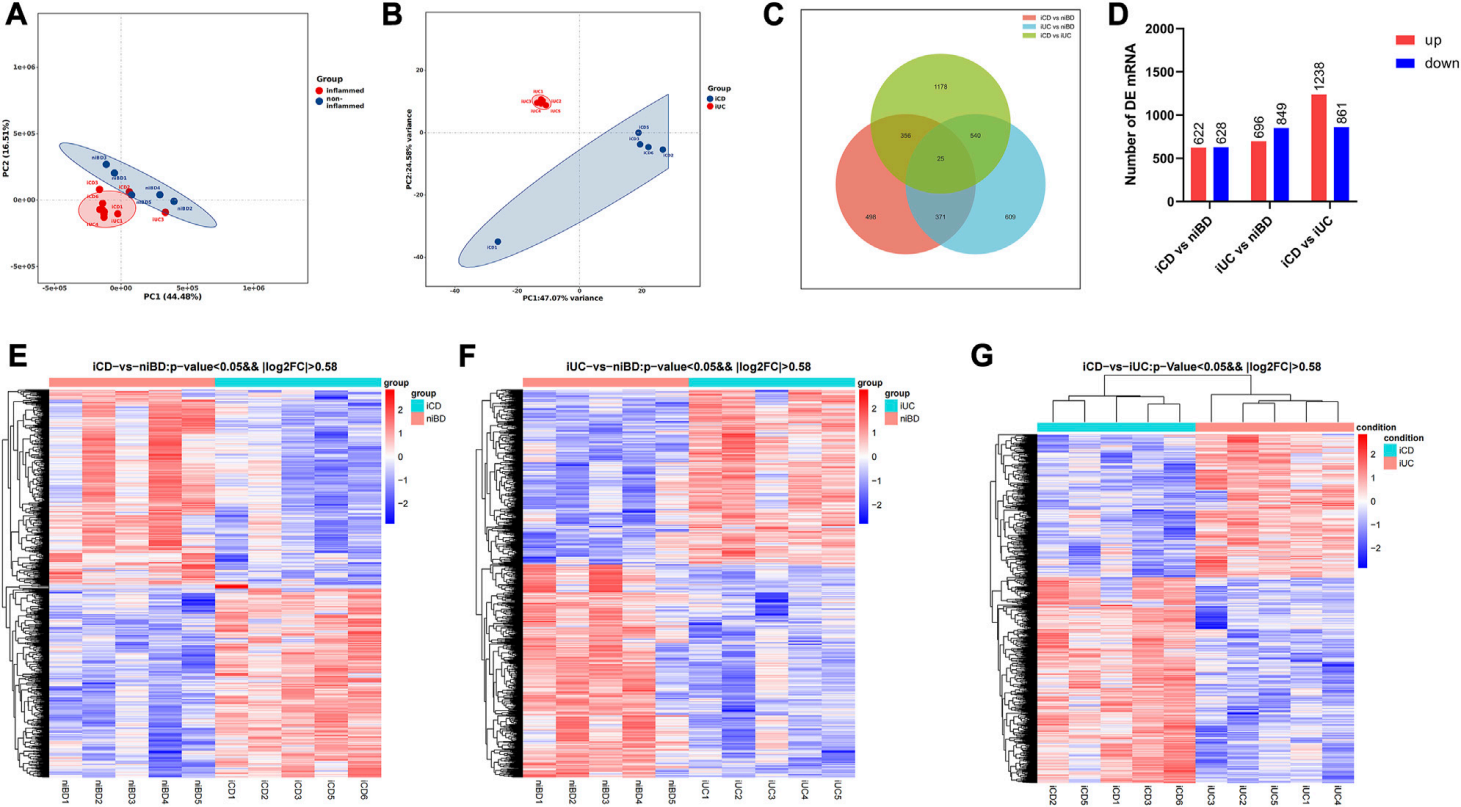
PCA and differential expression analysis of transcriptomic profiling. **(A,B)** Principal Component Analysis (PCA) plot displaying mRNA transcriptomic data. Each point represents a specific specimen, while each color represents a different group. Numbers along the perimeter indicate principal components (PC1-PC2), and numbers in parentheses represent the percentage variance accounted for by each PC. **(C)** Venn diagram illustrating the overlaps between differentially expressed mRNAs identified in iCD vs. niBD, iUC vs. niBD, and iCD vs. iUC comparisons. The criteria for differential expression were log2 fold change (log2FC) greater than absolute value of 0.58 and an adjusted *p*-value less than 0.05. **(D)** Bar graph showing the number of dysregulated mRNAs. Upregulated mRNAs are depicted in red, while downregulated mRNAs are shown in blue. The criteria for dysregulation were log2FC greater than absolute value of 0.58 and an adjusted *p*-value less than 0.05. **(E–G)** Heatmap representing differentially expressed mRNAs identified through whole transcriptomic analysis of IBD specimens. Each row corresponds to one mRNA, and each column represents a specimen. Upregulated and downregulated mRNAs are highlighted in red and blue, respectively. The dendrograms on top of the heatmap display hierarchical clustering correlation between the specimens.

Based on principal component analysis (PCA) of mRNA expressions, the results showed that approximately 44.48% and 16.51% of the variability in gene expression data were captured by the first and second principal components (PC1 and PC2), respectively (Figure 1A). This indicated that the non-inflamed specimens (niBD) formed a distinct cluster separate from the inflamed specimens (iCD and iUC). Furthermore, on PC1 and PC2, there was clear separation between iCD and iUC, accounting for 47.07% and 24.58% of the variability, respectively (Figure 1B). These findings suggest significant heterogeneity between iCD and iUC.

RNA transcript differential expression analysis of iCD, iUC, and niBD was performed after high-throughput RNA sequencing. The genes with fold-change (FC) > 1.5 and adjusted *p*-value <0.05 were considered differentially expressed genes (DEGs) with statistical significance. A total of 1250 and 1545 DEGs were identified from either iCD or iUC specimens compared to the niBD group (Figures 1C, D). Then, 2099 DEGs were identified when iCD was compared to iUC specimens (Figures 1C, D). To stratify the iCD, iUC, and niBD specimens, the expression profiles of DE mRNA (FC > 1.5) were compared through unsupervised hierarchical clustering. Compared to the niBD group, the heat map of these DE mRNAs showed intra-group similarity in the iCD or the iUC group (Figures 1E, F). Notably, the comparison of the iCD vs. iUC revealed a tight intra-group cluster and distinguished iCD from iUC (Figure 1G), indicating an underlying difference between iCD and iUC.

### 3.3 CD revealed dysregulation of enteric SMC apoptosis and proliferation

**FIGURE 2:**
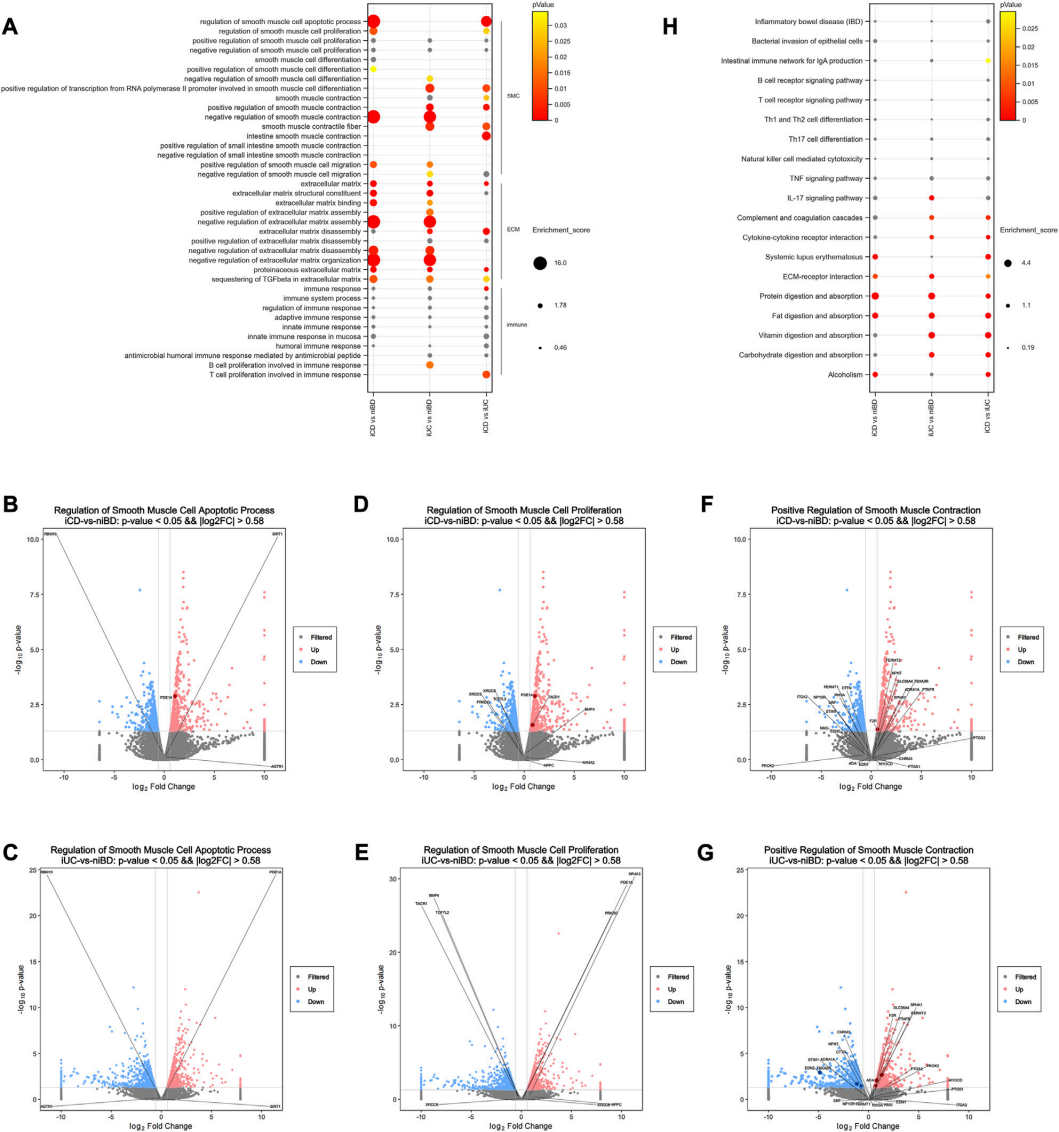
Pathway analysis of dysregulated mRNAs reveals dysregulated enteric smooth muscle cell apoptosis and proliferation in CD. **(A)** Selected Gene Ontology (GO) biological processes that exhibit significant enrichment among dysregulated mRNAs(log2FC >|0.58| and adjusted *p*-value <0.05). The enrichment score for each dysregulated gene annotated to the corresponding GO term is indicated. *p*-values greater than 0.05 are labeled in grey. **(B–G)** Volcano plots depicting dysregulated mRNAs. Pink and blue dots represent upregulated and downregulated mRNAs, respectively (log2FC >|0.58| and adjusted *p*-value <0.05). Non-significant mRNAs are displayed in grey. Dysregulated mRNAs associated with selected processes are labeled in dark red or blue. **(H)** Selected KEGG signaling pathways significantly enriched among dysregulated mRNAs (log2FC >|0.58| and adjusted *p*-value <0.05). The enrichment score for each dysregulated gene annotated to the corresponding KEGG pathway is presented. *p*-values greater than 0.05 are labeled in grey.

To identify disrupted biological processes and pathways in IBD patients, gene enrichment analysis was conducted to obtain overrepresented gene ontology (GO) terms of biological processes and Kyoto Encyclopedia of Genes and Genomes (KEGG) pathways from dysregulated genes. When GO terms were used, the two SMC-related terms, “regulation of SMC apoptotic process” and “regulation of SMC proliferation” were highly enriched in iCD but not in iUC (Figure 2A). Among the identified genes such as AGTR1, PDE1A, RBM10, SIRT1, BMP4, NPPC, NR4A3, PRKDC, TACR1, TCF7L2, XRCC5, and XRCC6, volcano plots revealed that PDE1A and TACR1 are the only two significantly upregulated mRNAs (log2FC >|0.58| and adjusted *p*-value <0.05) (Figures 2B–E). Similarly, “positive regulation of SMC contraction” was highly enriched in iUC but not in iCD; the genes involved (Figures 2F, G) were ADA, ADRA1A, CHRM3, CTTN, EDN1, EDN2, F2R, FERMT1, FERMT2, ITGA2, MYOCD, NMU, NPNT, NPY2R, PROK2, PTAFR, PTGS1, PTGS2, RHOA, SLC36A4, SPHK1, SRF, STIM1, and TBXA2R. These altered biological processes were overrepresented in direct comparison between the iCD and iUC groups (Figure 2A), which alludes to SMC phenotypic and subtle functional divergence between CD and UC. Notably, in extracellular matrix (ECM)- or immune-related terms, CD and UC presented similar enrichment patterns, except slight differences in biological processes of ECM, ECM disassembly, and proteinaceous ECM (Figure 2A).

When KEGG pathways were used, the pathways of bacterial invasion of epithelial cells, B or T cell receptor signaling, Th1/2/17 cell differentiation, natural killer cell-mediated cytotoxicity, TNF signaling, and IL-17 signaling did not show significant differences in iCD or iUC (Figure 2H). Interestingly, the activation of complement and coagulation cascades and cytokine-cytokine receptor interaction was observed significantly in iUC, whereas activation of systemic lupus erythematosus was predominant in iCD (Figure 2H). These activations were overrepresented in the direct comparison between the iCD and iUC groups (Figure 2H). Similar to the data obtained by the enrichment analysis of GO terms, ECM-receptor interaction pathway was activated in both iCD and iUC, albeit with slight differences (Figure 2H). In the pathways of protein/fat digestion and absorption, iCD and iUC presented similar activation but with slight variances; however, vitamin/carbohydrate digestion and absorption pathways were only activated in iUC (Figure 2H), suggesting that varying degrees of gastrointestinal mucosa injury have a differential impact on digestion and absorption between CD and UC. Moreover, the activation of “alcoholism” was enriched in iCD, not iUC (Figure 2H).

### 3.4 CD exhibited abnormalities in the enteric ANS

**FIGURE 3:**
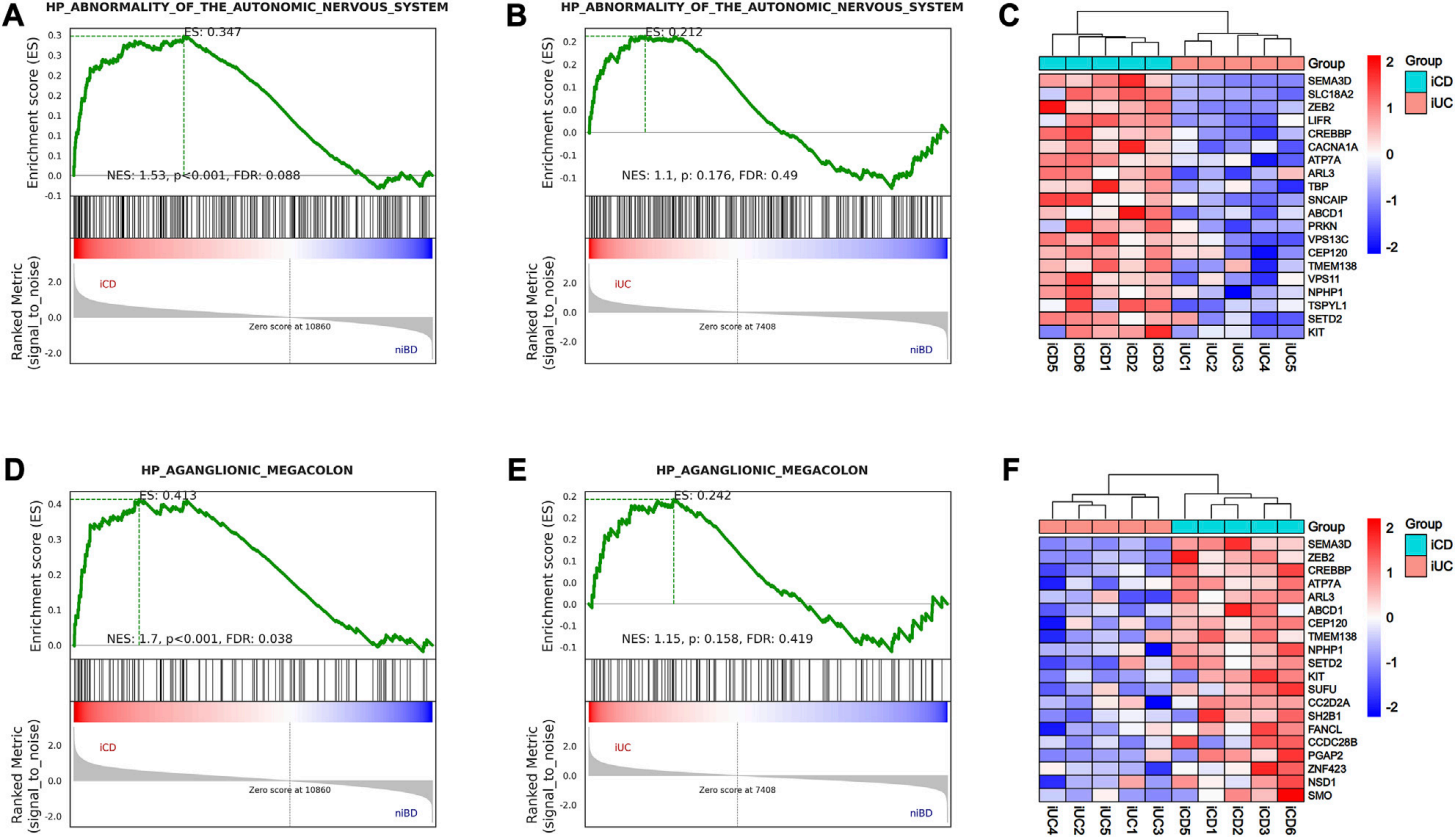
GSEA analysis shows activation of abnormality of the autonomic nervous system (ANS) and aganglionic megacolon in iCD. **(A,B)** Gene Set Enrichment Analysis (GSEA) of abnormality of the autonomic nervous system-related gene sets in iCD and iUC specimens vs. niBD. **(C)** Top 20 core enrichment genes associated with abnormality of the autonomic nervous system. **(D,E)** GSEA of aganglionic megacolon-related gene sets in iCD and iUC specimens vs. niBD. **(F)** Top 20 core enrichment genes associated with aganglionic megacolon.

The enteric nervous system (ENS) is a part of the ANS located in the digestive tract and innervating SMC with a marked influence on gastrointestinal function. Herein, we explored the ENS deviance in iCD and iUC using GSEA to identify the genes associated with abnormality of the autonomic nervous system and aganglionic megacolon. As a result, only iCD was significantly associated with the gene sets related to abnormality of the autonomic nervous system (Figures 3A, B) and aganglionic megacolon (Figures 3D, E), indicating that CD may involve pathogenic activity resembling ENS abnormalities or gangliopathy. Notably, SEMA3D emerged as the top-ranking gene among the top 20 core enrichment genes in both ANS abnormality (Figure 3C) and aganglionic megacolon (Figure 3F) gene sets.

### 3.5 Validating dysregulated genes SEMA3D and PDE1A: Implications for SMC and ANS dysfunction in CD

**FIGURE 4:**
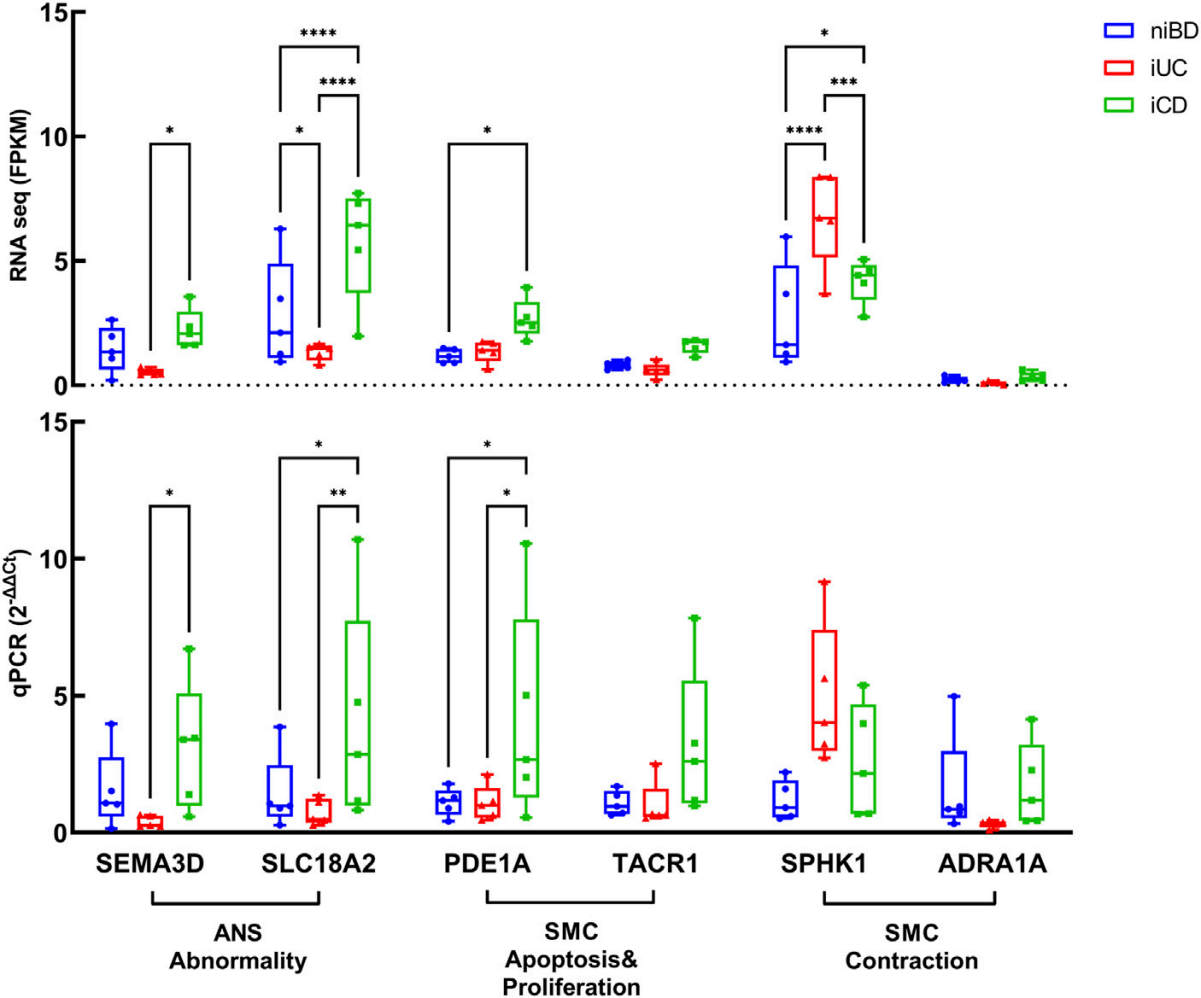
Validation by real-time qPCR of genes overactivated or downregulated in colon specimens of iCD (n = 5) and iUC (n = 5) compared to those from niBD (n = 5). Graphs display interleaved box and whisker plots representing the range from minimum to maximum values. For RNA sequence values expressed in FPKM and qPCR values expressed in 2^−ΔΔCt^, statistical analysis was performed using Fisher’s Least Significant Difference (LSD) test.Nonsignificant *p*-values (>0.05) are denoted. Asterisks (*) indicate statistical significance levels: *p ≤ 0.05, **p ≤ 0.01, ***p ≤ 0.001, ****p ≤ 0.0001.

We conducted validation of dysregulated key genes involved in smooth muscle cell (SMC) apoptosis and proliferation, as well as abnormalities in the autonomic nervous system (ANS), using quantitative polymerase chain reaction (qPCR). The results confirmed that SEMA3D (iUC vs. iCD, *p* = 0.0309) and SLC18A2 (iUC vs. iCD, *p* = 0.0087), which were identified as core enrichment genes for ANS abnormality, were upregulated by 8-fold and 5-fold respectively in iCD (Figure 4). Additionally, the core enrichment gene PDE1A, implicated in SMC apoptosis and proliferation, exhibited a 4-fold upregulation in iCD (iUC vs. iCD, *p* = 0.0144) (Figure 4). Conversely, SPHK1 was upregulated in iUC for positive regulation of SMC contraction (niBD vs. iUC, *p* = 0.0030) (Figure 4).

The dysregulation of key DEGs at the protein levels detected in iCD and iUC (already confirmed at the mRNA level by RNAseq and real-time qPCR) was assessed by immunohistochemistry (IHC) staining. The clinical information of specimen sections is provided in Supplementary Table S6.

**FIGURE 5:**
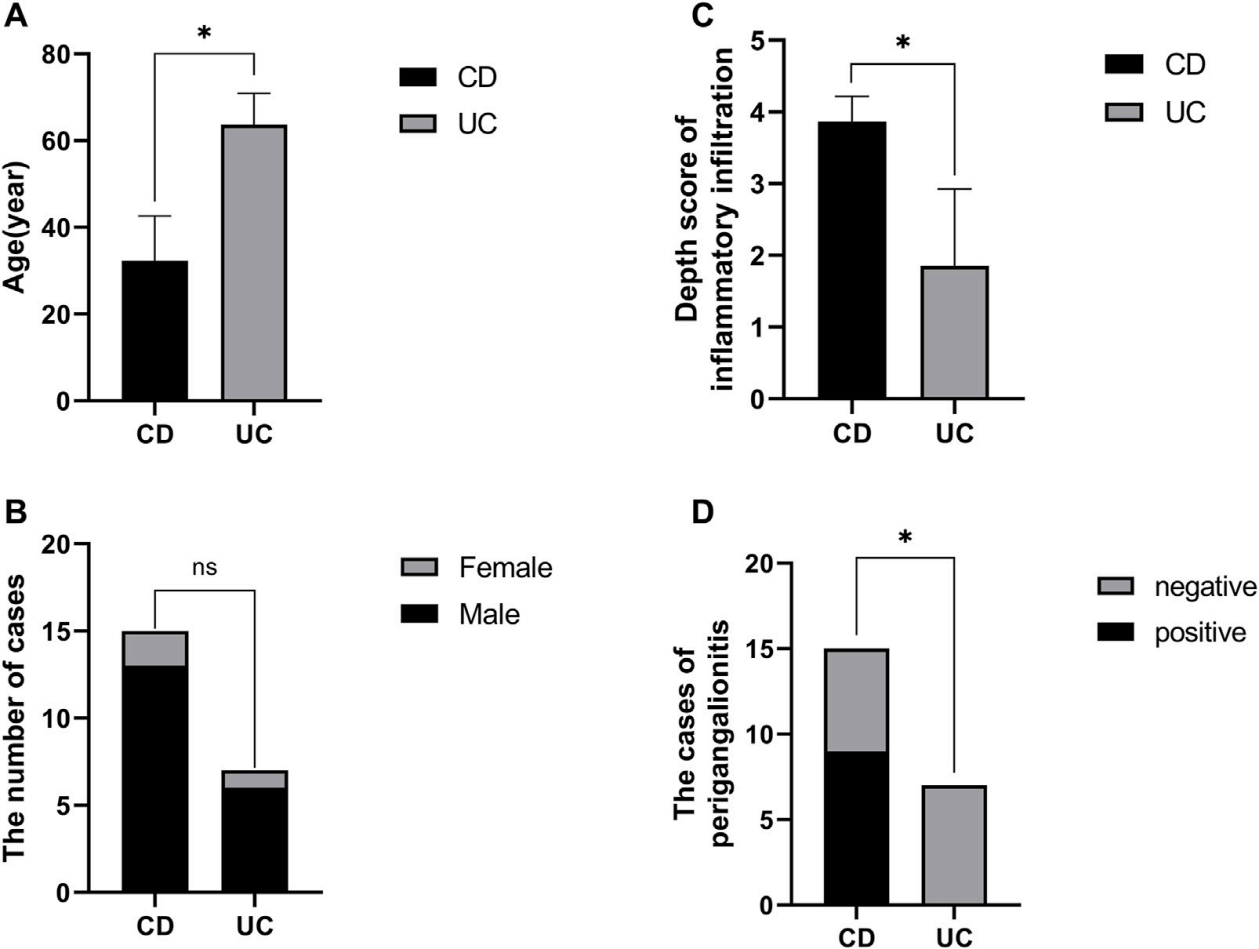
Baseline information of specimen sections prepared with non-inflamed and inflamed colon from CD and UC patients. **(A)** Comparison of disease onset ages between CD and UC patients. **(B)** Gender distribution in CD and UC patients. **(C)** Variation in depth scores of inflammatory infiltration observed in CD and UC patients. **(D)** Proportion of CD patients exhibiting periganglionitis.

**FIGURE 6:**
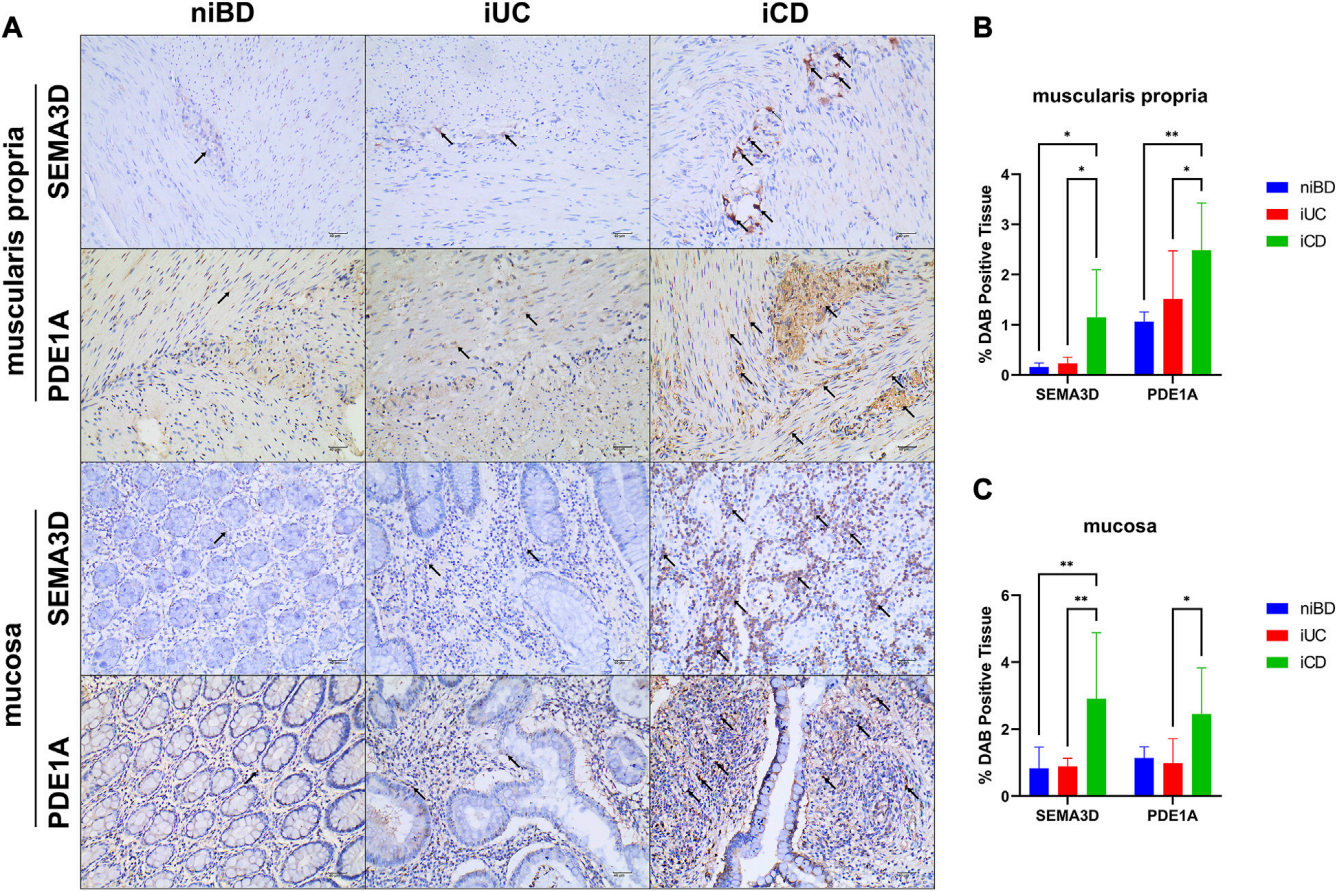
Abundant expression of SEMA3D and PDE1A proteins in layers of muscularis propria and mucosa in inflamed CD specimens. **(A)** Immunohistochemistry (IHC) images showing representative colon specimens from patients with CD and UC, including non-inflammatory and inflammatory samples. Staining demonstrates expression of SEMA3D and PDE1A at a magnification of ×20. The scale bar corresponds to 40 μm. **(B,C)** Graphs presenting the quantification of staining (percentage of DAB-positive tissue) in niBD, iUC, and iCD cohorts (niBD, n = 4; iUC, n = 7; iCD, n = 15). Statistical analysis was performed using Fisher’s LSD test. Nonsignificant *p*-values (>0.05) are denoted. Asterisks (*) indicate statistical significance levels: *p ≤ 0.05, **p ≤ 0.01.

In total, we subjected 15 specimens from iCD, 7 specimens from iUC, and 4 non-inflammatory specimens from niBD (2 niCD +2 niUC) to IHC staining. PDE1A, associated with SMC apoptosis and proliferation, and SEMA3D, associated with ANS abnormality were selected for IHC staining. Consistent with the data presented in Table 2, CD patients tended to have a early onset age (Figure 5A), histologically higher depth score of inflammatory infiltration (Figure 5C), and perigangalionitis (iCD vs. iUC, *p* = 0.0167) (Figure 5D) (Supplementary Figure S2). The IHC data demonstrated that SEMA3D protein levels were upregulated in muscularis propria (iUC vs. iCD, *p* = 0.0178) (Figures 6A, B) and mucosal layer (iUC vs. iCD, *p* = 0.0023) of iCD (Figures 6A, C), and displayed significant aggregation around the ganglia in iCD (Figure 6A). Additionally, the protein level of PDE1A was significantly increased in muscularis propria (iUC vs. iCD, *p* = 0.0128) (Figures 6A, B) and mucosa (iUC vs. iCD, *p* = 0.0243) layers of iCD (Figures 6A, C).

Overall, these findings validated the mRNA data obtained in this study and suggest the role of SEMA3D and PDE1A as key genes involved in the dysregulation of SMC apoptosis and proliferation, as well as in orchestrating the abnormality of the enteric ANS, particularly in the pathogenesis of CD.

## 4 Discussion

This study revealed a widespread distinguishable dysregulation of mRNA expression between CD and UC in the colon inflammatory region. The regulation of SMC apoptosis and proliferation was significantly enriched in iCD, rather than in iUC. The involved PDE1A gene was upregulated 4-fold and 1.5-fold in iCD, as assessed by qPCR and IHC, respectively. Moreover, iCD was significantly associated with gene sets of ANS abnormality, while SEMA3D gene was upregulated 8-fold and 5-fold, respectively, compared to iUC.

In previous studies, the phenomenon of smooth muscle hyperplasia/hypertrophy in CD has been described briefly (Suekane et al., 2010; Flynn et al., 2011; Scirocco et al., 2013). A recent novel histological grading scheme study demonstrated that muscularization, including hypertrophy of the MP and smooth muscle hyperplasia of the SM, is the most prevalent histological change in CD (Chen et al., 2017), accompanied by neuronal hypertrophy in both myenteric (Auerbach’s) plexuses and submucosal (Meissner’s) plexuses (Chen et al., 2017).

In this study, CD was significantly associated with the biological processes of SMC apoptosis and proliferation regulation. Both mRNA and protein levels of PDE1A involved in enteric SMC apoptosis/proliferation balance increased significantly in iCD. Cyclic nucleotide phosphodiesterases (PDEs) are critical in the homeostasis of cyclic nucleotides that regulate SMC growth by hydrolyzing cAMP or cGMP. Previous findings (Nagel et al., 2006) suggested that cytoplasmic PDE1A is associated with the “contractile” phenotype, whereas nuclear PDE1A is associated with the “synthetic” phenotype. Decreasing the levels of nuclear PDE1A via RNA interference or pharmacological inhibition significantly attenuated SMC growth by reducing proliferation via G1 arrest, induced apoptosis, elevated intracellular cGMP level, and altered gene expression, which was consistent with growth arrest and apoptosis (Nagel et al., 2006). Conversely, cytoplasmic PDE1A regulates myosin light chain phosphorylation with little effect on apoptosis (Nagel et al., 2006). In another study (Rajagopal et al., 2015), PDE1A expression was induced and accompanied by an increase in PDE1A activity in muscle cells isolated from muscle strips cultured with IL-1 β (interleukin-1 beta) or TNF- α (tumor necrosis factor alpha) or obtained from the colon of TNBS (2,4,6-trinitrobenzene sulfonic acid)-treated mice. Also, nitric oxide-induced muscle relaxation was inhibited in longitudinal muscle cells. This inhibition was completely reversed by the combination of both 1400 W dihydrochloride and vinpocetine (a PDE1 inhibitor) (Rajagopal et al., 2015). The inhibition of smooth muscle relaxation during inflammation reflected the combined effects of decreased sGC activity via S-nitrosylation and increased cGMP hydrolysis via PDE1 expression, thereby indicating that PDE1A might be a novel target for relieving altered pathogenesis of enteric smooth muscle in CD (Rajagopal et al., 2015).

Previously, 9 patients with both Hirschsprung disease (HSCR, also called congenital aganglionic megacolon) and IBD were described, suggesting an association between the two conditions (Sherman et al., 1989). HSCR is a neurocristopathy caused by a failure of the ENS progenitors derived from neural crest cells (NCCs) to migrate, proliferate, differentiate, or survive on and within the gastrointestinal tract, resulting in aganglionosis in the colon. This association has been confirmed in a few case reports and small case series (Levin et al., 2012; Kim and Kim, 2017). A recent population-based cohort study showed that individuals with HSCR had a 5-fold higher risk for IBD than those without HSCR (Löf Granström et al., 2018). Also, a follow-up study (Granström et al., 2021) found that the extent of aganglionosis is related to the risk of IBD. This theory was also proposed in a meta-analysis (Nakamura et al., 2018), including 14 studies encompassing a total of 66 patients with HSCR associated with IBD; moreover, the distribution of IBD is in 72.3% of CD patients. Another population-based cohort study (Bernstein et al., 2021) showed that individuals diagnosed with HSCR resulted in a 12-fold increased risk of subsequently diagnosed IBD. Interestingly, IBD can emerge in >2% of patients with HSCR and is more frequently classified as CD rather than UC (Bernstein et al., 2021).

In the present study, CD was significantly associated with gene sets of abnormality of ANS and aganglionic megacolon, indicating that the abnormality of ANS/ENS, such as gangliopathy may show pathogenic activity in CD similar to that in HSCR. Both the mRNA and protein levels of SEMA3D involved in the abnormality of ANS and aganglionic megacolon increased significantly in iCD. SEMA3D encodes a member of the semaphorin III family of secreted signaling proteins involved in axon guidance during neuronal development and is one of the three signaling pathways of HSCR pathogenesis (Luzón-Toro et al., 2013; Jiang et al., 2015; Kapoor et al., 2015); the other two are RET and EDNRB signaling pathways (Amiel et al., 2008; Tilghman et al., 2019).

SEMA3D has been implicated in the development of HSCR and contributes to risk in European (Luzón-Toro et al., 2013; Jiang et al., 2015; Kapoor et al., 2015) and Asian ancestries (Wang et al., 2011; Li et al., 2017; Gunadi et al., 2020). In a previous study (Luzón-Toro et al., 2013), the E198K-SEMA3D, A131T-SEMA3A, and S598G-SEMA3A mutations presented an increased protein level in the smooth muscle layer of ganglionic segments. Moreover, A131T-SEMA3A also maintained high protein levels in the aganglionic muscle layers. The coincident upregulation of SEMA3A expression in aganglionic colons was detected in Chinese patients of HSCR: the circular muscle layer, the submucosa, and the longitudinal muscles layer (Wang et al., 2011). These findings indicated that the SEMA3 variants increase the SEMA3 proteins levels in the HSCR colon tissue, thus supporting the functional implication of SEMA3s as a signaling molecule to influence the phenotype of HSCR patient. Thus, SEMA3D involvement of ANS/ENS abnormality may be a common pathogenesis mechanism in CD and HSCR.

However, it is important to acknowledge several limitations in our study. Firstly, future investigations should consider using a larger sample size to enhance the statistical power of our analysis. While the presence of variation in clinical and demographic characteristics may have constrained our analysis, it is worth noting that the observed alterations in RNA expression most likely arise from the underlying disease pathophysiology. This inference is supported by the fact that most of the variations in the clinical and demographic characteristics of the specimens were not statistically significant, except for onset age, lesion location, and inflammatory infiltration depth, which have traditionally been considered disease phenotypic features. To evaluate the potential influence of colonic location on RNA expression levels, we compared the expressions of PDE1A, SEMA3D, and SLC18A2 between non-inflamed whole-wall cecum tissues (n = 6) and non-inflamed whole-wall transverse (n = 6) and descending (n = 6) colonic tissues, as depicted in Supplementary Figure S3. Our analysis did not reveal any significant differences in RNA expressions among the different colonic locations. Therefore, it could be cautiously inferred that the disparities in PDE1A, SEMA3D, and SLC18A2 expression levels among iCD, iUC, and niBD may reflect the inflammation status or disease phenotypic features rather than the anatomical location. Secondly, it is important to note that our study samples consisted exclusively of individuals of Chinese ethnicity. Consequently, future investigations should aim to explore the genetic backgrounds of different ethnic groups to obtain a more comprehensive understanding. Thirdly, although our transcriptome profile suggests abnormalities in enteric autonomic nervous system (ANS) and dysregulation of enteric smooth muscle cell (SMC) apoptosis/proliferation in the inflamed colon of CD, further research is necessary to determine whether these biological processes are secondary to the “inflammation-smooth muscle hyperplasia axis,” analogous to chronic asthma, or if they involve independent pathways.

Conclusively, this study highlights the presence of ANS abnormality and dysregulation of SMC apoptosis/proliferation in the pathogenesis of CD. The identified genes, including SEMA3D and PDE1A, may serve as potential diagnostic biomarkers for differentiating between CD and UC, as well as therapeutic targets for restoring enteric dysautonomia and SMC proliferative disorders in CD. Future diagnostic and therapeutic strategies could be designed based on the dysregulation of enteric SMC apoptosis and proliferation, as well as enteric dysautonomia.

## Data Availability

The datasets presented in this study can be found in online repositories. The names of the repository/repositories and accession number(s) can be found below: https://www.ncbi.nlm.nih.gov/geo/, GSE227747.
