# Supplementary material for: Transcriptome profiling of intact bowel wall reveals that PDE1A and SEMA3D are possible markers with roles in enteric smooth muscle apoptosis, proliferative disorders, and dysautonomia in Crohn’s disease
Source: Front Genet. 2023 Aug 31;14:1194882. doi: 10.3389/fgene.2023.1194882 (PMC10505932; doi:10.3389/fgene.2023.1194882)
Supplement: Supplementary file 1 [file DataSheet1.docx]

Supplementary Material

Transcriptome Profiling of Intact Bowel Wall Reveals that PDE1A and SEMA3D are Possible Markers with Roles in Enteric Smooth Muscle Apoptosis, Proliferative Disorders, and Dysautonomia in Crohn's Disease.

Yun Yang, Lin Xia, Wenming Yang, Ziqiang Wang, Wenjian Meng, Mingming Zhang, Qin Ma, Junhe Gou, Junjian Wang, Ye Shu*, Xiaoting Wu*

*** Correspondence:** Xiaoting Wu: wxt1@medmail.com.cn

Ye Shu: [sy999222@hotmail.com](mailto:sy999222@hotmail.com)

# Supplementary Figures and Tables

## Supplementary Tables

**Supplementary Table 1.** **Gene set of aganglionic megacolon**

| ABCD1 | ASCL1 | CBY1 | CSPP1 | FANCA | FOXF1 | KATNIP | MKKS | PGAP3 | RAD51C | SETBP1 | SUFU | TYR |
| --- | --- | --- | --- | --- | --- | --- | --- | --- | --- | --- | --- | --- |
| ACTG2 | ATP7A | CC2D2A | DDX59 | FANCB | GATA1 | KIAA0586 | MKS1 | PHOX2B | RET | SETD2 | TBX1 | UBE2T |
| AHI1 | ATRX | CCDC28B | DHCR7 | FANCC | GDNF | KIFBP | MYO1H | PIBF1 | RFWD3 | SF3B4 | TCF4 | UFD1 |
| APC2 | B9D1 | CEP104 | ECE1 | FANCD2 | GJB2 | KIT | NAA10 | PIGL | RMRP | SH2B1 | TCTN1 | XRCC2 |
| ARL13B | BBS1 | CEP120 | EDN3 | FANCE | GJB6 | KITLG | NPHP1 | PIGN | RPGRIP1L | SLC6A8 | TCTN2 | ZEB2 |
| ARL3 | BCOR | CEP290 | EDNRB | FANCF | GP1BB | KRAS | NRTN | PIGO | RREB1 | SLX4 | TMEM138 | ZNF423 |
| ARL6 | BDNF | CEP41 | EP300 | FANCG | HIRA | L1CAM | NSD1 | PIGV | SALL4 | SMO | TMEM216 |  |
| ARMC9 | BRCA1 | COMT | ERBB2 | FANCI | HYLS1 | MAD2L2 | PALB2 | PIGW | SEC24C | SNAI2 | TMEM231 |  |
| ARVCF | BRCA2 | CPLANE1 | ERBB3 | FANCL | INPP5E | MBTPS2 | PAX3 | PIGY | SEMA3C | SOX10 | TMEM237 |  |
| ARX | BRIP1 | CREBBP | ERCC4 | FANCM | JMJD1C | MITF | PGAP2 | RAD51 | SEMA3D | SREBF1 | TMEM67 |  |

(<http://www.gsea-msigdb.org/gsea/msigdb/geneset_page.jsp?geneSetName=HP_AGANGLIONIC_MEGACOLON&keywords=aganglionic%20megacolon>)

**Supplementary Table 2.** **Gene set of abnormality of autonomic nervous system**

| AAAS | ATP7A | CBY1 | CYP11B2 | FANCD2 | GJB2 | KCNJ5 | MKS1 | PIGV | RPGRIP1L | SLC6A2 | TCF4 | UFD1 |
| --- | --- | --- | --- | --- | --- | --- | --- | --- | --- | --- | --- | --- |
| ABCD1 | ATRX | CC2D2A | DBH | FANCE | GJB6 | KCNQ1 | MMEL1 | PIGW | RREB1 | SLC6A8 | TCTN1 | VPS11 |
| ACOX1 | ATXN2 | CCDC28B | DDX59 | FANCF | GLA | KIAA0586 | MYO1H | PIGY | SALL4 | SLX4 | TCTN2 | VPS13A |
| ACTG2 | ATXN3 | CCT5 | DEPDC5 | FANCG | GLI2 | KIF1B | NAA10 | PINK1 | SCN10A | SMC1A | TDGF1 | VPS13C |
| ADH1C | ATXN8OS | CDON | DHCR7 | FANCI | GLUD2 | KIFBP | NODAL | PLA2G6 | SCN11A | SMO | TGIF1 | VPS35 |
| AHI1 | B2M | CEP104 | DISP1 | FANCL | GMPPA | KIT | NOS1AP | PODXL | SCN3A | SNAI2 | TMEM138 | WDR45 |
| AKAP9 | B9D1 | CEP120 | DLL1 | FANCM | GP1BB | KITLG | NPHP1 | POLR3A | SCN4B | SNCA | TMEM216 | WFS1 |
| ANK2 | BBS1 | CEP290 | DNAJC13 | FBXO7 | GSN | KRAS | NR4A2 | POU2AF1 | SCN5A | SNCAIP | TMEM231 | XRCC2 |
| APC2 | BCOR | CEP41 | DNAJC6 | FGF8 | HEXB | L1CAM | NRTN | PPOX | SCN9A | SNTA1 | TMEM237 | ZEB2 |
| ARL13B | BDNF | CHCHD2 | ECE1 | FGFR1 | HIRA | LEP | NSD1 | PRKN | SEC24C | SOX10 | TMEM67 | ZIC2 |
| ARL3 | BRAT1 | CHRNA3 | EDN3 | FMR1 | HTRA2 | LEPR | NTRK1 | PRNP | SEMA3C | SPG11 | TNFSF15 | ZNF423 |
| ARL6 | BRCA1 | CISD2 | EDNRB | FOXF1 | HYLS1 | LGI1 | PALB2 | PSAP | SEMA3D | SPIB | TNPO3 |  |
| ARMC9 | BRCA2 | COL1A1 | EIF4G1 | FOXH1 | IL12A | LIFR | PARK7 | PTCH1 | SETBP1 | SPTLC1 | TRDN |  |
| ARSA | BRIP1 | COL5A1 | ELP1 | GABBR2 | IL12RB1 | LMNB1 | PAX3 | RAD21 | SETD2 | SPTLC2 | TSPYL1 |  |
| ARVCF | CACNA1A | COL5A2 | EP300 | GAS1 | INPP5E | LRRK2 | PGAP2 | RAD51 | SF3B4 | SREBF1 | TTR |  |
| ARX | CACNA1C | COMT | ERBB2 | GATA1 | IRF5 | MAD2L2 | PGAP3 | RAD51C | SH2B1 | STAG2 | TUBB3 |  |
| ASCL1 | CALM1 | COQ2 | ERBB3 | GBA | JMJD1C | MAPT | PHOX2B | RELN | SHH | SUFU | TWNK |  |
| ATL1 | CALM2 | CPLANE1 | ERCC4 | GBE1 | KATNIP | MBTPS2 | PIBF1 | RET | SIM1 | SYNJ1 | TXN2 |  |
| ATL3 | CALM3 | CREBBP | FANCA | GCK | KCNE1 | MECP2 | PIGL | RFC1 | SIX3 | TBP | TYR |  |
| ATP1A2 | CAV1 | CSPP1 | FANCB | GDNF | KCNE2 | MITF | PIGN | RFWD3 | SLC18A2 | TBX1 | UBE2T |  |
| ATP1A3 | CAV3 | CYB561 | FANCC | GIGYF2 | KCNH2 | MKKS | PIGO | RMRP | SLC1A3 | TBX5 | UCHL1 |  |

(<http://www.gsea-msigdb.org/gsea/msigdb/geneset_page.jsp?geneSetName=HP_ABNORMALITY_OF_THE_AUTONOMIC_NERVOUS_SYSTEM&keywords=autonomic%20nerve>)

**Supplementary Table 3. The main reagents for immunohistochemistry**

| **Item** | **manufacturer** | **Cat.No** | **Dilution ratio** |
| --- | --- | --- | --- |
| Absolute ethanol | Chengdu Haixing Chemical Reagent Factory | GB678-90 | 75%, 85%, 95% |
| Xylene | Tianjin Zhiyuan Chemical Reagent Co., LTD. | 202150101 | working solution |
| Citrate buffer | ZSGB Biotechnology | ZLI-9065 | 0.01mol/L |
| Hematoxylin dyeing solution | J&K Scientific | LM10N13 | working solution |
| Neutral gum | Labgic Technology | BL704A | working solution |
| PBS buffer | ZSGB Biotechnology | ZLI-9062 | 0.01mol/L |
| Hydrogen peroxide | Sinopharm | 011092708 | 3% |
| DAB Kit | ZSGB Biotechnology | ZLI-9018 | 1:20 |
| Normal goat serum | BOSTER Biological Technology | AR1009 | 1:9 |
| Primary antibody (SEMA3D) | NOVUS | NBP1-85517 | 1:50 |
| Primary antibody (PDE1A) | Proteintech | 12442-2-AP | 1:200 |
| Secondary antibody | ZSGB Biotechnology Co., LTD. | SP9001 | working solution |

**Supplementary Table 4. Demographic and clinical information of individual patient**

|  | **Crohn's Disease (CD)** | | | | | | **Ulcer Colitis (UC)** | | | | | |
| --- | --- | --- | --- | --- | --- | --- | --- | --- | --- | --- | --- | --- |
| **Patient No.** | CD1 | CD2 | CD3 | CD4 | CD5 | CD6 | UC1 | UC2 | UC3 | UC4 | UC5 | UC6 |
| Patient characteristics |  |  |  |  |  |  |  |  |  |  |  |  |
| Age (year) | 32 | 23 | 24 | 23 | 46 | 25 | 65 | 75 | 67 | 56 | 67 | 54 |
| Gender | M | M | M | M | M | M | M | M | M | M | M | F |
| Smoker |  | √ |  |  |  |  | √ |  | √ |  | √ |  |
| Alcohol |  |  |  |  |  |  | √ |  | √ | √ | √ |  |
| Preoperative treatment history |  |  |  |  |  |  |  |  |  |  |  |  |
| 5-ASA |  |  | √ | √ | √ | √ | √ | √ | √ | √ | √ | √ |
| Steroids |  |  |  | √ | √ | √ |  | √ |  | √ | √ | √ |
| Immunomodulation | √ |  |  | √ |  |  |  | √ |  | √ |  |  |
| Anti-TNF |  |  |  | √ |  | √ |  |  |  |  |  |  |
| Non-anti-TNF biologic treatment |  |  |  |  |  |  |  |  |  |  |  |  |
| Location involvement |  |  |  |  |  |  |  |  |  |  |  |  |
| Ileum | √ | √ | √ |  | √ | √ |  |  |  |  |  |  |
| Cecum | √ | √ | √ |  |  |  |  |  |  |  |  |  |
| Ascending colon | √ |  | √ |  |  | √ | √ | √ |  | √ | √ | √ |
| Transverse colon | √ |  | √ | √ |  |  | √ | √ | √ | √ | √ | √ |
| Descending colon |  |  |  | √ |  |  | √ | √ | √ | √ | √ | √ |
| Sigmoid |  |  |  | √ | √ |  | √ | √ |  | √ | √ | √ |
| Rectal |  |  |  |  |  |  | √ | √ |  | √ | √ | √ |
| Phenotypes |  |  |  |  |  |  |  |  |  |  |  |  |
| Depth score of inflammatory infiltration*: | 4 | 3 | 4 | 3 | 4 | 4 | 3 | 1 | 1 | 1 | 3 | 3 |
| Acute inflammatory |  | √ |  |  | √ |  | √ | √ | √ |  |  | √ |
| Chronic inflammatory | √ | √ | √ | √ | √ | √ |  | √ | √ | √ | √ | √ |
| Ulcers |  |  | √ |  | √ |  | √ | √ |  | √ | √ | √ |
| Penetrate/fistula | √ |  |  |  | √ |  |  |  |  |  |  |  |
| Stricturing |  | √ |  | √ | √ | √ |  |  |  |  |  |  |
| Perigangliitis | √ |  |  |  |  | √ |  |  |  |  |  |  |
| Postoperative outcome |  |  |  |  |  |  |  |  |  |  |  |  |
| Biologic use |  | √ |  |  | √ | √ |  |  |  |  |  |  |
| Median time to first resection(months) | 96 | 24 | 6 | 60 | 120 | 20 | 0.5 | 120 | 6 | 48 | 24 | 120 |
| Median time from first resection to second resection(months) | NA | NA | NA | NA | NA | 70 | NA | NA | NA | NA | NA | NA |

*Depth score of inflammatory infiltration:
mucosa: Score 1; muscularis mucosa(MM): Score 2; submucosa(SM): Score 3; muscularis propria(MP): Score 4; subserosal adventitia(SS): Score 5

**Supplementary Table 5.** Grouping design

| **Group** | **Specimen No.** | **From Patient No.** | **Specimen Location** |
| --- | --- | --- | --- |
| iCD | iCD1 | CD1 | Inflamed transverse colon |
|  | iCD2 | CD2 | Inflamed cecum |
|  | iCD3 | CD3 | Inflamed transverse colon |
|  | iCD4 | CD4 | Inflamed transverse colon |
|  | iCD5 | CD5 | Inflamed sigmoid |
|  | iCD6 | CD6 | Inflamed ascending colon |
| iUC | iUC1 | UC1 | Inflamed transverse colon |
|  | iUC2 | UC2 | Inflamed transverse colon |
|  | iUC3 | UC3 | Inflamed transverse colon |
|  | iUC4 | UC4 | Inflamed transverse colon |
|  | iUC5 | UC5 | Inflamed transverse colon |
|  | iUC6 | UC6 | Inflamed transverse colon |
| niBD | niBD1 | CD1 | Non-inflamed descending colon |
|  | niBD2 | CD2 | Non-inflamed transverse colon |
|  | niBD3 | CD3 | Non-inflamed descending colon |
|  | niBD4 | UC4 | Non-inflamed cecum |
|  | niBD5 | UC5 | Non-inflamed cecum |
|  | niBD6 | UC6 | Non-inflamed cecum |

**Supplementary Table 6.** Clinical information of tissue slides

| **Group** | **Patient No.** | **Gender** | **Age** | **Biopsy method** | **Tissue Integrity** | **Inflammatory status** |
| --- | --- | --- | --- | --- | --- | --- |
| iCD | CD1 | Male | 32 | surgical excision | intact bowel wall | Inflamed |
|  | CD2 | Male | 23 | surgical excision | intact bowel wall | Inflamed |
|  | CD3 | Male | 24 | surgical excision | intact bowel wall | Inflamed |
|  | CD4 | Male | 23 | surgical excision | intact bowel wall | Inflamed |
|  | CD5 | Male | 46 | surgical excision | intact bowel wall | Inflamed |
|  | CD6 | Male | 25 | surgical excision | intact bowel wall | Inflamed |
|  | CD7 | Male | 24 | surgical excision | intact bowel wall | Inflamed |
|  | CD8 | Male | 28 | surgical excision | intact bowel wall | Inflamed |
|  | CD9 | Male | 33 | surgical excision | intact bowel wall | Inflamed |
|  | CD10 | Male | 48 | surgical excision | intact bowel wall | Inflamed |
|  | CD11 | Male | 32 | surgical excision | intact bowel wall | Inflamed |
|  | CD12 | Male | 30 | surgical excision | intact bowel wall | Inflamed |
|  | CD13 | Male | 58 | surgical excision | intact bowel wall | Inflamed |
|  | CD14 | Female | 30 | surgical excision | intact bowel wall | Inflamed |
|  | CD15 | Female | 28 | surgical excision | intact bowel wall | Inflamed |
| iUC | UC1 | Male | 65 | surgical excision | intact bowel wall | Inflamed |
|  | UC2 | Male | 75 | surgical excision | intact bowel wall | Inflamed |
|  | UC3 | Male | 67 | surgical excision | intact bowel wall | Inflamed |
|  | UC4 | Male | 56 | surgical excision | intact bowel wall | Inflamed |
|  | UC5 | Male | 67 | surgical excision | intact bowel wall | Inflamed |
|  | UC6 | Female | 54 | surgical excision | intact bowel wall | Inflamed |
|  | UC7 | Male | 62 | surgical excision | intact bowel wall | Inflamed |
| niBD | CD1 | Male | 32 | surgical excision | intact bowel wall | Non-inflamed |
|  | CD2 | Male | 23 | surgical excision | intact bowel wall | Non-inflamed |
|  | UC4 | Male | 56 | surgical excision | intact bowel wall | Non-inflamed |
|  | UC5 | Male | 67 | surgical excision | intact bowel wall | Non-inflamed |

## Supplementary Figures

**
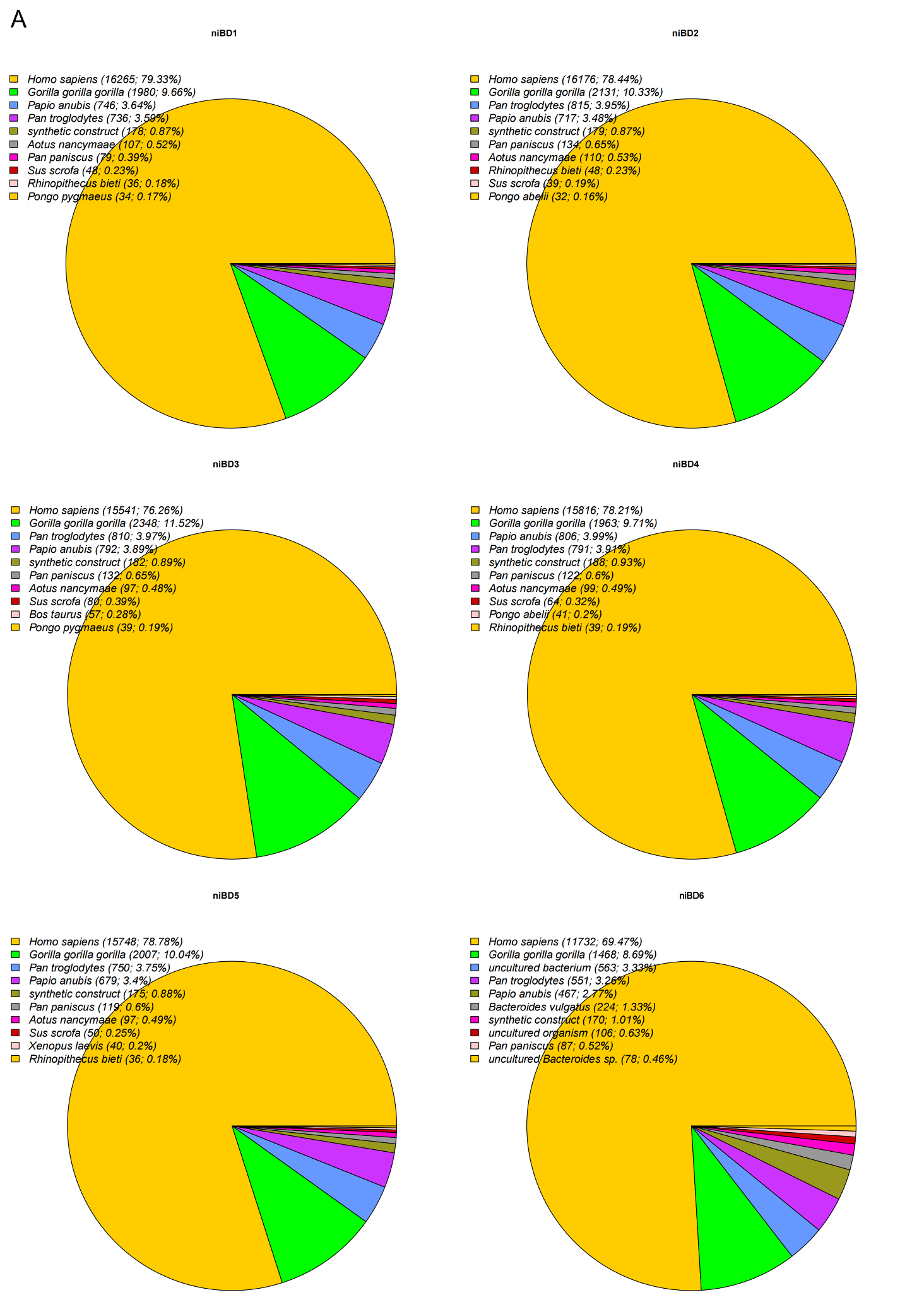
**


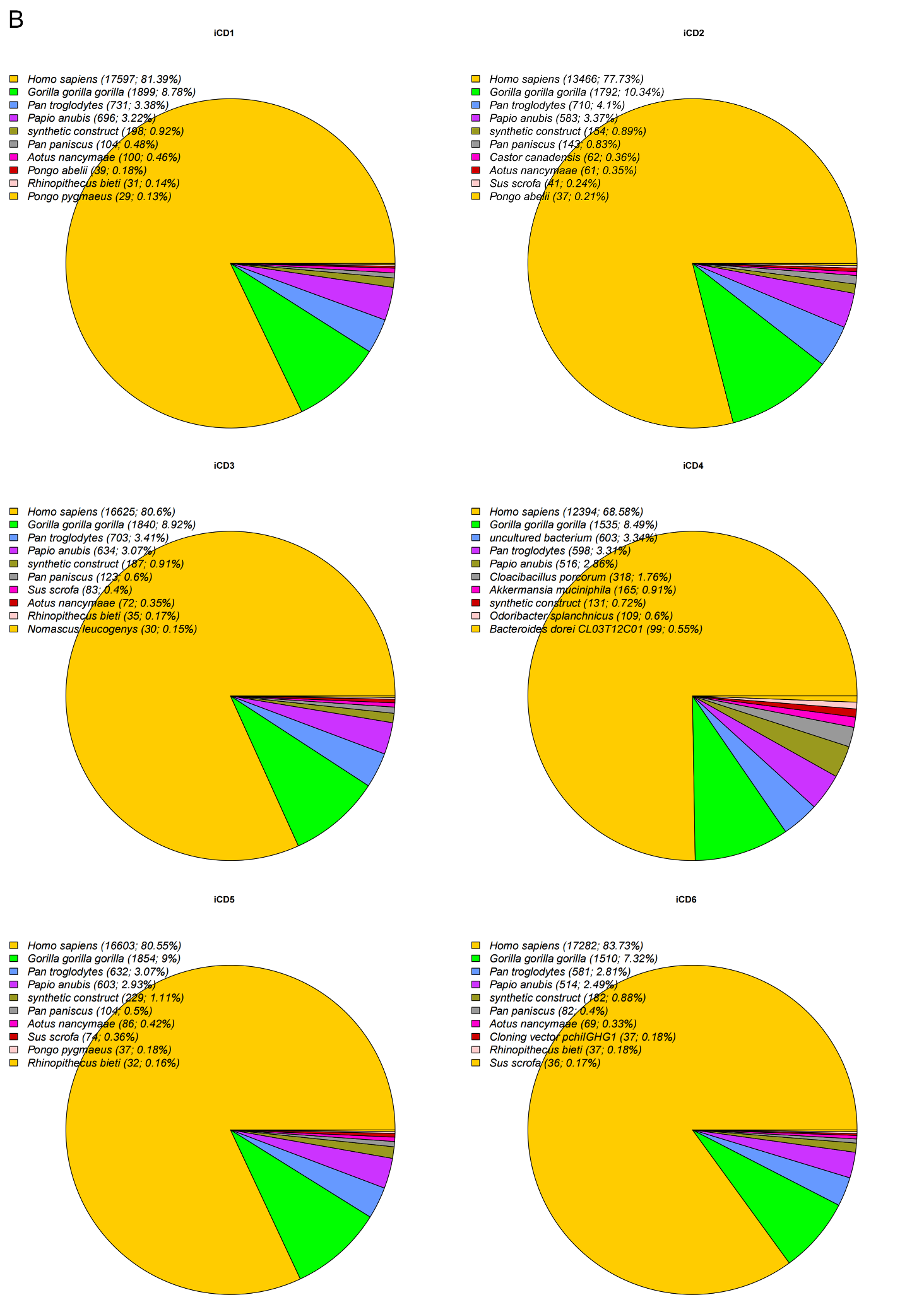

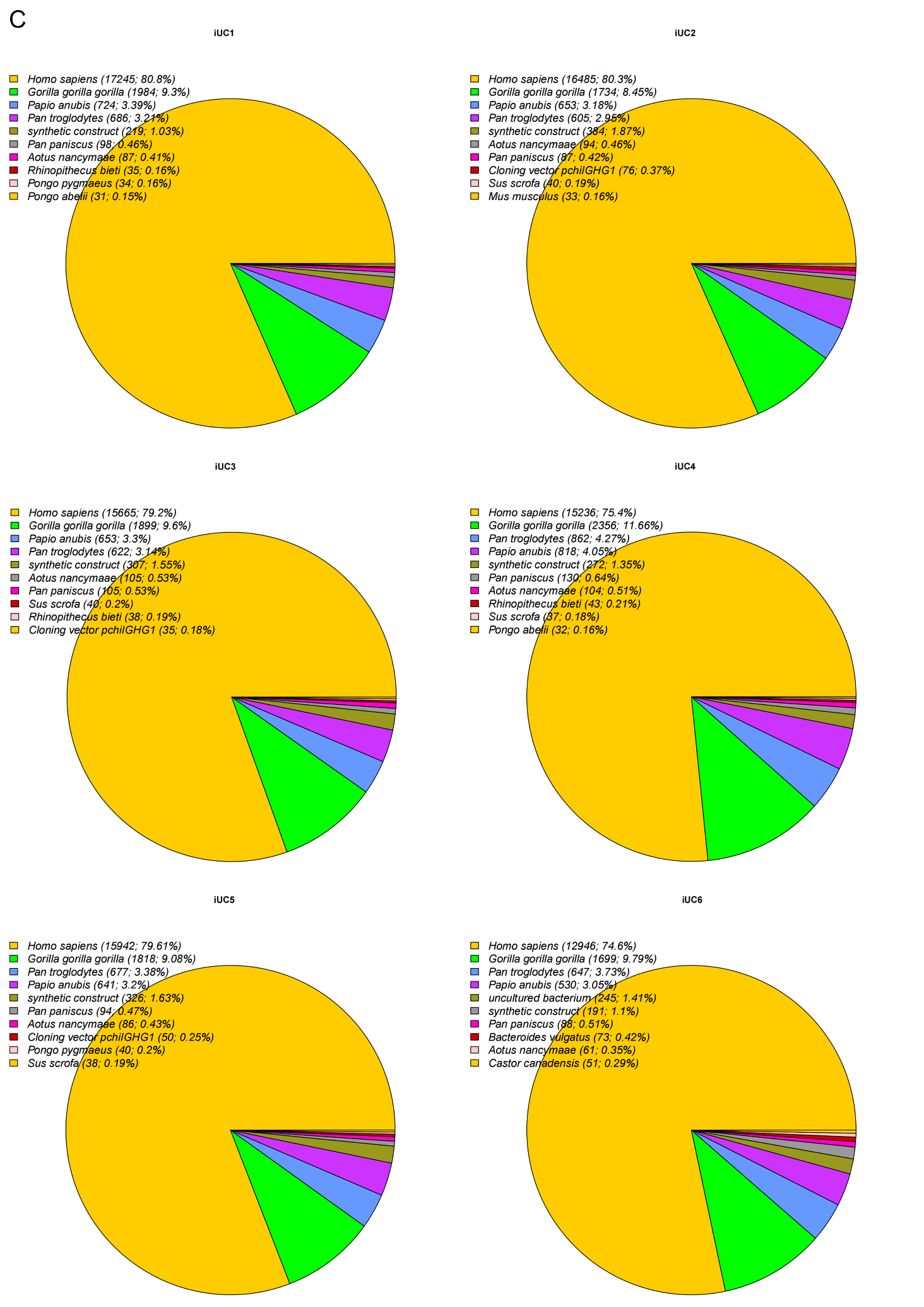


**Supplementary Figure 1.** Three samples (niBD6 (A), iCD4 (B) and iUC6 (C) niBD6 (A), iCD4 (B) and iUC6 (C)) were found to have different degrees of bacterial RNA contamination during sequencing, and two of them(niBD6 and iCD4) were found the contaminating bacterial RNA exceed over 5%. Therefore, in order to prevent bias caused by bacterial contamination, we excluded these three samples from the subsequent bioinformatics analysis.


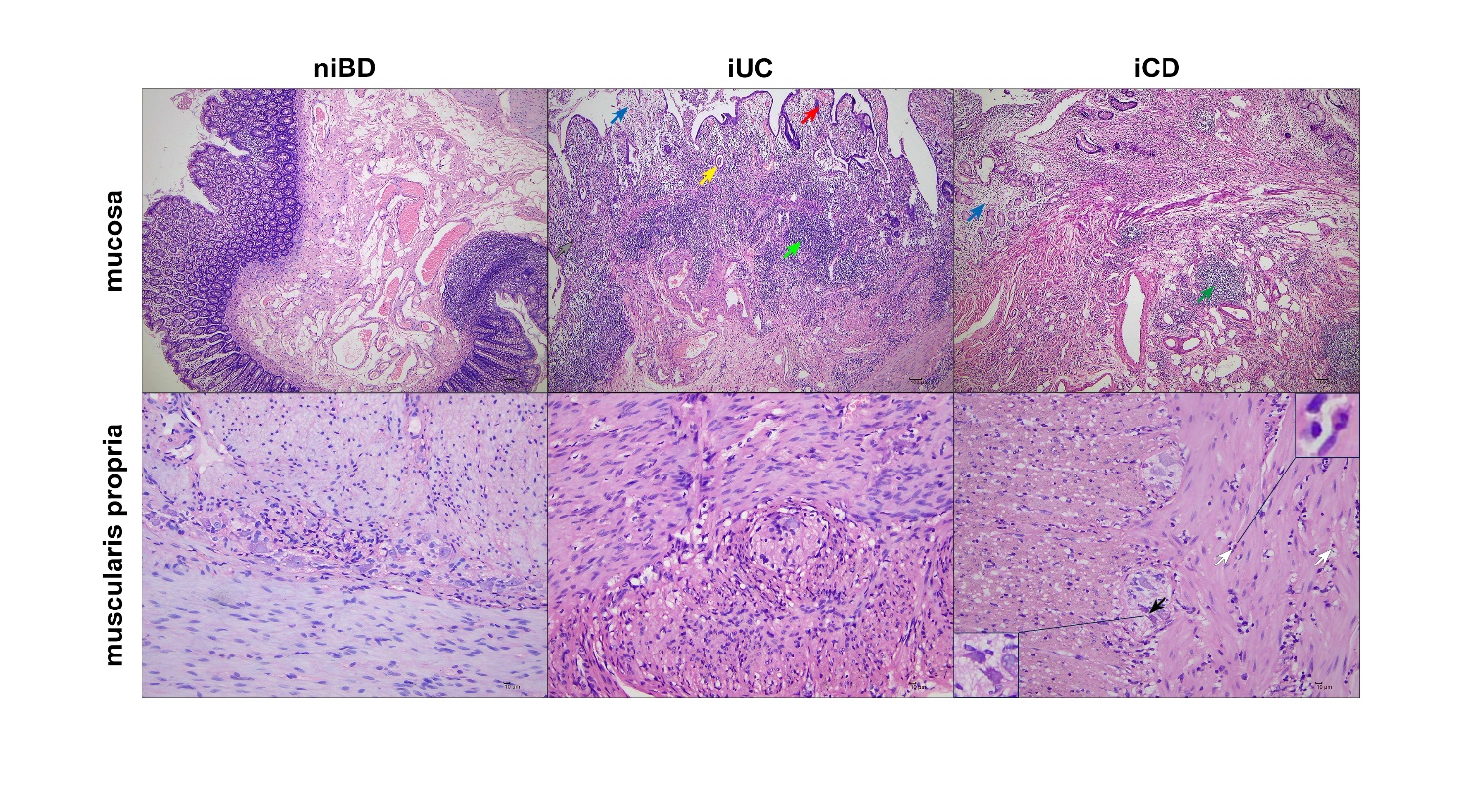


**Supplementary Figure 2.** Morphologically, UC is characterized by superficial inflammation that is confined to the mucosal and submucosal layers with the presence of mucosal edema (blue arrow), massive necrosis (grey arrow), crypt abscesses (yellow arrow), inflammatory cell infiltration (light green arrow), telangiectasia congestion (red arrow). Unlike UC, a transmural pattern of inflammation is a hallmark of CD, with the presence of lymphoid follicle aggregation (dark green arrow), polymorphonuclear neutrophils infiltration (white arrow), and neuron degeneration and necrosis (black arrow).


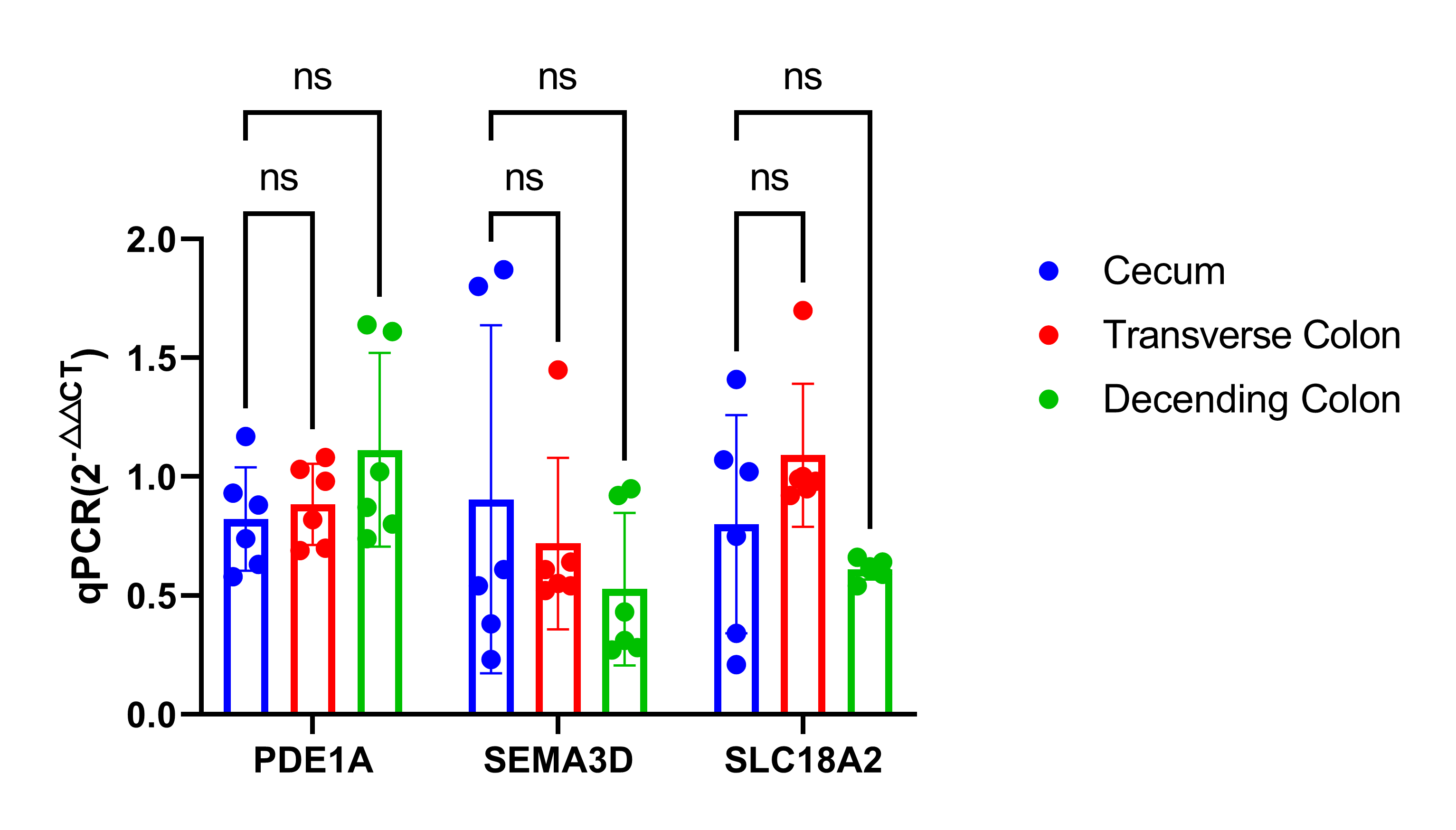


**Supplementary Figure 3: Comparison of RNA expression levels of PDE1A, SEMA3D, and SLC18A2 among different colonic locations.**

The figure presents interleaved box and whisker plots illustrating the mean values with standard deviation. The comparison was made between non-inflamed whole-wall cecum tissues (n=6) and non-inflamed whole-wall transverse (n=6) and descending (n=6) colonic tissues. The RNA expressions were measured using quantitative PCR (qPCR) and were expressed as 2^-ΔΔCt^. Statistical analysis was performed using Fisher's Least Significant Difference (LSD) test. Non-significant P-values (>0.05) are denoted. Asterisks (*) denote statistical significance levels: *P ≤ 0.05, **P ≤ 0.01, ***P ≤ 0.001, ****P ≤ 0.0001.
